# Evaluation of routine four-quadrant re-excision in patients undergoing breast-conserving surgery

**DOI:** 10.1590/1806-9282.20252235

**Published:** 2026-07-10

**Authors:** Cemal Hacıalioğlu, Hasan Ediz Sıkar, Bağış Taşdoğan, Yasin Tosun, Hasan Fehmi Küçük

**Affiliations:** 1 Istanbul Kartal Dr. Lutfi Kirdar City Hospital, General Surgery Department – Istanbul, Turkey.

**Keywords:** Breast-conserving surgery, Re-excision, Local recurrence

## Abstract

**OBJECTIVE:**

The aim of this study was to evaluate the contribution of routine four-quadrant re-excision performed during breast-conserving surgery in detecting de novo tumor foci and ensuring negative surgical margins in patients with breast cancer.

**METHODS:**

A retrospective analysis was conducted on 44 female patients who underwent breast-conserving surgery between April 2021 and April 2023. In all cases, routine re-excision was performed in the superior, inferior, medial, and lateral quadrants during the same operative session. Based on pathological findings, patients with additional tumor foci in the re-excision specimens were classified as Group 1, while those without were classified as Group 2.

**RESULTS:**

The mean age of the patients was 54.2±11.6 years, with an average follow-up period of 21.5±8.3 months. Additional tumor foci were identified in seven patients (15.9%). There were no statistically significant differences between the groups in terms of age, tumor size, hormone receptor status, or histopathological characteristics (p>0.05).

**CONCLUSION:**

Routine four-quadrant re-excision may aid in the identification of microscopic de novo tumor foci even when clear surgical margins are achieved. However, larger multicenter studies with longer follow-up periods are warranted to determine its potential impact on local recurrence and long-term outcomes.

## INTRODUCTION

Breast cancer is the most frequently diagnosed malignancy among women and remains one of the leading causes of cancer-related mortality worldwide. According to GLOBOCAN 2020 data, approximately 2.3 million new cases and 685,000 deaths are reported annually^1^.

Breast-conserving surgery (BCS) has become increasingly preferred, particularly for patients with early-stage breast cancer, as it provides survival rates comparable to mastectomy while offering superior cosmetic outcomes and improved quality of life^2,3^. However, the major drawback of BCS is its relatively higher rate of local recurrence compared with mastectomy^4^. One of the main causes of local recurrence is positive surgical margins, which necessitate re-operation in approximately 10–30% of patients^5,6^.

Several strategies have been developed to reduce this problem. One of these involves the routine excision of cavity wall tissues surrounding the tumor during BCS. This technique, referred to in the literature as cavity-shave margins, has been shown in several randomized controlled trials and meta-analyses to significantly decrease the rates of positive surgical margins and re-operation^7,8^. Although cavity-shave margins have been widely described in the literature, the surgical approach used in our study differs slightly from the classical cavity shaving technique. In our practice, systematic re-excision was performed in four predefined quadrants (superior, inferior, medial, and lateral) surrounding the tumor cavity during the same operative session. Nevertheless, whether this method should be routinely implemented in all centers remains a matter of debate, and data from different geographic regions are crucial in shaping clinical decision-making.

This study aims to evaluate the role of routine four-quadrant re-excision performed during the same operative session in patients undergoing BCS for breast cancer, particularly in detecting additional tumor foci and contributing to negative margin achievement. Furthermore, the study investigates the potential of this approach to identify de novo tumor foci that may not be detected by contemporary imaging modalities.

## METHODS

This study was designed as a retrospective cohort analysis and was approved by the Kartal Dr. Lütfi Kırdar State Hospital ethics committee on 08.03.2023 with the number 2023/514/245/6. Between April 2021 and April 2023, female patients who underwent BCS for breast cancer in our clinic were included. Only patients with postoperative pathological confirmation of invasive breast carcinoma and stage I–II disease were eligible for the study.

Male breast cancer cases; patients with multicentric tumors detected on preoperative imaging; those with non-malignant postoperative pathology; individuals with positive or close (<2 mm) surgical margins in the primary specimen; patients with stage III–IV disease; and those who had received neoadjuvant therapy were excluded.

In all patients included in the study, routine four-quadrant re-excision (superior, inferior, medial, and lateral) was performed during the same operative session following primary tumor excision. The surfaces of the re-excised specimens facing the tumor cavity were marked with metallic clips and sent for histopathological examination.

Based on the pathological evaluation, patients in whom the re-excision biopsy revealed additional foci of invasive carcinoma or in situ carcinoma were classified as Group 1, while those without any additional tumor foci were classified as Group 2.

A total of 44 patients were included in the study. All patients underwent preoperative ultrasonography and positron emission tomography-computed tomography (PET-CT), and mammography was additionally performed in 40 patients (90.9%). In our institution, PET-CT is frequently used as part of the staging protocol to evaluate possible regional or distant metastases, particularly in patients referred from external centers with previously established imaging findings or when there is clinical suspicion of more advanced disease.

Postoperative pathological evaluations were conducted independently by two pathologists with specific expertise in breast cancer.

Statistical analyses were performed using SPSS software (version 27.0; IBM Corp., Armonk, NY, USA). Descriptive data were expressed as mean±standard deviation, median, frequency, and percentage values. For quantitative variables showing a normal distribution, comparisons between groups were made using the independent-samples t-test, whereas non-parametric tests were applied for data that did not meet normality assumptions. Qualitative variables were compared using Pearson’s chi-square and Fisher’s exact tests. A p-value of <0.05 was considered statistically significant.

## RESULTS

A total of 44 female patients were included in the study. The mean age of the patients was 54.2±11.6 years (range, 32–74 years). The mean follow-up period was calculated as 21.5±8.3 months (range, 7–37 months). Additional tumor foci were detected in seven patients (15.9%) within the surgical re-excision specimens (Group 1), whereas no additional foci were identified in 37 patients (84.1%), designated as Group 2 (Table 1).

**Table 1:** Comparison of demographic characteristics, preoperative findings, and follow-up durations between groups.

	Group 1 (n: 7)	Group 2 (n: 37)	p
Age (years)	54±9.7	52.2±12.01	0.956
Mammography (n)	7 (100%)	33 (89.2%)	0.487
Ultrasonography (n)	7 (100%)	37 (100%)	-
Preoperative tumor size (mm)	20±7.32	19.08±7.08	0.756
PET-CT axillary involvement	0 (0%)	1 (2.3%)	0.841
Follow-up duration (months)	16.85±9.58	22.37±7.87	0.107

PET-CT: positron emission tomography-computed tomography.

Comparisons between the two groups revealed no statistically significant differences in terms of age, mammography performance rate, axillary involvement on PET-CT, or preoperative tumor size (p>0.05).

Postoperative pathological findings also revealed no statistically significant differences between the two groups (Table 2). There were no significant differences in histologic grade, nuclear grade, hormone receptor positivity (estrogen receptor [ER], progesterone receptor [PR]), HER2 status, Ki-67 index, tumor size, presence of an in situ component, number of sentinel lymph nodes, or axillary dissection rates (p>0.05). In Group 1, the size of additional tumor foci ranged from 4 to 13 mm (Table 3). None of the additional tumor foci detected in the re-excision specimens was classified as invasive lobular carcinoma. The additional lesions consisted of invasive ductal carcinoma and ductal carcinoma in situ (DCIS).

**Table 2:** Comparison of postoperative pathological findings between groups.

	Group 1 (n: 7)	Group 2 (n: 37)	P
Histological grade	1.71±0.75	2.02±0.68	0.283
Nuclear grade	1.85±0.69	2.24±0.59	0.133
Estrogen receptor positive	7 (100%)	30 (81.1%)	0.269
Progesterone receptor positive	7 (100%)	28 (75.7%)	0.175
HER2 positive	2 (28.6%)	21 (56.8%)	0.170
Ki-67 (%)	22.42±7.61	28.67±21.85	0.186
Postoperative tumor size (mm)	21.57±5.82	22.91±9.82	0.729
In situ component present (n)	6 (85.7%)	23 (62.2%)	0.227
Sentinel lymph nodes (n)	3.57±0.78	3.7±1.46	0.82
Positive sentinel nodes (n)	0.42±0.78	0.35±0.67	0.788
Axillary dissection performed (n)	2 (28.6%)	5 (13.5%)	0.307

HER2: human epidermal growth factor receptor 2; Ki-67: Ki-67 proliferation index.

**Table 3:** Postoperative pathological findings in Group 1.

Patient No.	Positive re-excision site	Lesion size (mm)	Additional focus type	In situ grade in the primary specimen
1.	Lateral	13	Invasive	Intermediate
2.	Inferior	11	Invasive	High
3.	Lateral	4	DCIS	No in situ component identified in the primary specimen
4.	Inferior	10	Invasive	High
5.	Superior and lateral	4 and 5	DCIS	Intermediate
6.	Medial and inferior	3 and 0.5	DCIS	Intermediate
7.	Inferior	10	Invasive	Intermediate

DCIS: ductal carcinoma in situ.

## DISCUSSION

Since the first identification of the ER and the routine introduction of hormonal therapy agents such as tamoxifen, additional biomarkers including the progesterone receptor, HER2, Ki-67, and tumor microenvironment-related factors such as tumor-infiltrating lymphocytes have emerged as key determinants in guiding treatment decisions^9,10^. In addition, recent studies have emphasized that diagnostic pathways and clinicopathological characteristics may vary according to molecular subtypes and demographic factors, further influencing individualized breast cancer management^9^. The traditional TNM classification system, which relies on tumor size, lymph-node involvement, and metastasis status, has gradually evolved with the incorporation of immunohistochemical and genetic parameters, leading to refined molecular subtyping of breast cancer^11,12^.

In addition, screening-related factors such as breast density continue to influence the diagnostic performance of conventional imaging modalities and may contribute to the underdetection of occult breast lesions^13^. In terms of surgical evolution, the pivotal MILAN trial, which first demonstrated the feasibility of avoiding mastectomy, laid the foundation for the transition from mastectomy to lumpectomy, now recognized as BCS. Consequently, the number of patients undergoing total mastectomy has declined substantially in recent years^3^.

The evolution of BCS has not been limited to surgical advancements alone but has also been closely linked to the improvement of local control through the integration of radiotherapy^14^. While earlier studies emphasized the importance of maintaining wide surgical margins, current evidence supports the adoption of narrower margins for invasive lesions, highlighting that margin status remains a critical determinant in preventing local recurrence^15^.

Having negative surgical margins wider than 1 mm significantly reduces the rate of local recurrence. A recent meta-analysis reported that margins wider than 2 mm were associated with a 50% reduction in local recurrence rates^16^. These findings demonstrate that extending surgical margins is an effective strategy for minimizing the risk of recurrence. In our study, all patients had surgical margins at least 2 mm from the tumor in the primary specimen, which eliminated the potential confounding effect of close margins on local recurrence risk and ensured standardized comparison between groups.

Despite the advances in systemic therapies with increasing tumor-suppressive and cytotoxic efficacy, local recurrence after BCS remains a significant clinical challenge^17^. The relatively small sample size (n=44) limits the statistical power of subgroup analyses and should be considered when interpreting the findings. In particular, the absence of statistically significant differences between groups should not be interpreted as evidence of equivalence, as the study may be underpowered to detect clinically meaningful differences, especially given the small number of patients in Group 1.

In clinical practice, particularly in patients with the Luminal A subtype, who generally have a favorable prognosis and can achieve adequate systemic control with hormonal therapy alone, difficulties in maintaining local control continue to pose concerns for both patients and treating physicians.

During the developmental phase of BCS, particular emphasis was placed on the therapeutic potential of radiotherapy in eradicating possible in situ foci that might remain along the surgical margins. Indeed, local recurrence rates were significantly higher in patient groups who did not receive radiotherapy compared with those who underwent adjuvant radiotherapy after BCS^2^.

A meta-analysis of 17 randomized controlled trials involving 10,801 women demonstrated that postoperative radiotherapy following BCS reduced local recurrence rates by approximately 50%^18^. More recent series have reported local recurrence rates ranging between 2.5 and 5.5% among patients who received adjuvant radiotherapy after BCS^17,19^.

In daily clinical practice, the presence of in situ foci within the tumor bed or along the surgical margins alone is insufficient to fully explain local recurrences. Although several studies have investigated which patient subgroups are at a higher risk for local recurrence, not all patients exhibit outcomes consistent with these predictive patterns^17,19^. In cases where close or positive surgical margins are identified during secondary pathological evaluation following the initial operation, early re-excision or mastectomy is typically planned. Similarly, patients who experience local recurrence are managed according to comparable surgical treatment protocols.

Local recurrence rates following BCS have been reported to be higher than those observed after mastectomy, and positive surgical margins have been identified as one of the major contributing factors^4,5^. The need for a second surgical procedure due to margin positivity not only increases patient morbidity but also delays the initiation of adjuvant treatments^6^. Therefore, developing intraoperative strategies that can ensure negative margins during BCS holds great clinical significance.

In our study, the outcomes of routine four-quadrant re-excision performed during the same operative session were evaluated in 44 patients who underwent BCS. Additional tumor foci were detected in seven patients (15.9%) within the re-excision specimens. This finding suggests that microscopic tumor foci may persist despite negative surgical margins and that standard excision alone may be insufficient for their complete removal. Comparable results have been reported in the literature. For instance, in the randomized controlled trial conducted by Chagpar et al., the rate of detecting additional tumor foci in the cavity-shave group was 19%, and the re-operation rate was significantly reduced^7^. Similarly, several meta-analyses have demonstrated that cavity-shaving can reduce the rates of positive margins and re-operation by approximately 30–50%^20^. The detection of in situ and invasive tumor foci in re-excision specimens, despite negative surgical margins in patients who underwent routine re-excision, supports the hypothesis that local recurrences may originate from de novo tumor foci within the residual breast tissue. From a biological perspective, experimental studies have demonstrated that even microscopic residual tumor foci may retain proliferative potential through complex apoptotic and microenvironment-related mechanisms, potentially contributing to disease persistence^21^.

In our study, no statistically significant differences were observed between the groups in terms of age, tumor size, histologic subtype, hormone receptor status, HER2 expression, or proliferative index. This finding indicates that the presence of additional tumor foci cannot be reliably predicted based on clinical or histopathological parameters. Similarly, previous studies have reported no strong correlation between the presence of additional foci and patient age, tumor size, or molecular subtype^22^. Therefore, predicting the presence of occult tumor foci solely on the basis of preoperative risk factors appears to be challenging. However, the exclusion of patients who had received neoadjuvant chemotherapy in our cohort may have influenced this observation.

A detailed analysis of Group 1 patients revealed that four had intermediate-grade and two had high-grade in situ carcinoma in the primary specimen; in situ carcinoma at the tumor periphery was observed in only two of these cases. Notably, in one patient, no in situ carcinoma was identified in the primary specimen, yet a 4×2 mm in situ carcinoma focus was detected in the re-excision specimen.

Performing re-excision during the same operative session allowed for the identification of multifocal tumor foci in Group 1 patients, despite negative surgical margins. The absence of any parameters associated with an increased risk of local recurrence in either group suggests that routine re-excision may play a potential role in detecting de novo tumor foci in patients with early-stage breast cancer.

However, whether these microscopic foci can be reliably identified through contemporary imaging modalities remains open to discussion.

In Turkey, national screening guidelines currently recommend biennial mammography for women aged over 40 years. In younger patients, ultrasonography is commonly used as a complementary imaging modality, particularly when mammography yields BI-RADS 0 findings. Although breast magnetic resonance imaging (MRI) is increasingly employed in clinical practice, it is not yet a routine part of screening protocols.

In our study, all patients older than 40 years underwent mammography, while ultrasonography was routinely performed in those under 40 years. No additional tumor foci were identified on preoperative imaging in any of the cases. The lack of MRI evaluation in our clinical workflow represents an important limitation of this study. Nevertheless, previous studies have consistently shown that conventional imaging modalities such as mammography and ultrasonography are insufficient in detecting multifocality and multicentricity, and that even advanced imaging techniques such as MRI may not always detect small or clinically occult lesions^23,24^. The detection of additional foci measuring 4–13 mm in our cohort supports these findings.

Therefore, the potential contribution of MRI beyond standard imaging modalities warrants further investigation in future studies.

The clinical significance of our findings lies in providing real-world evidence from a single-center cohort in Turkey, demonstrating that routine four-quadrant re-excision can enhance the detection of additional tumor foci during BCS. In healthcare settings where intraoperative frozen-section evaluation is not routinely available, implementing such simple and reproducible surgical strategies may help minimize the risk of residual disease. While routine re-excision might modestly increase operative time and excised tissue volume, potentially affecting cosmetic outcomes, its capacity to reduce re-operation rates remains a substantial advantage^25^.

This study has several limitations, including a relatively small sample size, its single-center design, and a short follow-up duration. The mean follow-up period of 21.5 months is insufficient to accurately assess local recurrence rates. Therefore, no conclusions can be drawn regarding the impact of routine re-excision on local recurrence based on the present findings.

Nevertheless, the detection of additional tumor foci in 15.9% of patients during this early period suggests that routine re-excision may contribute to improved detection of residual disease.

In conclusion, our findings suggest that routine four-quadrant re-excision during BCS may facilitate the detection of additional microscopic tumor foci that may remain undetected after standard excision. However, the present study was not designed to evaluate the impact of this approach on local recurrence, re-operation rates, cosmetic outcomes, or surgical complications. Therefore, the clinical implications of these findings should be interpreted with caution, and further large-scale, prospective studies are required to clarify the potential benefits of this approach.

## CONCLUSION

In our study, additional tumor foci were detected in re-excision specimens even among patients who achieved negative surgical margins after BCS. This finding highlights that modern imaging modalities remain insufficient to identify all microscopic tumor extensions, and that routine re-excision may represent a potential surgical strategy to address this limitation.

Further large-scale, multicenter studies with extended follow-up are warranted to evaluate the long-term effects of this approach on local recurrence and overall oncologic outcomes.

## Data Availability

The datasets generated and/or analyzed during the current study are available from the corresponding author upon reasonable request.
